# Molecular Assays on Cutaneous Swabs as an Effective, Non-Invasive Diagnostic Technique for Cutaneous Leishmaniasis: Results from a Retrospective Study Conducted in Italy

**DOI:** 10.3390/tropicalmed10060158

**Published:** 2025-06-09

**Authors:** Anna Barbiero, Andrea Aiello, Nunziata Ciccone, Simona Pollini, Francesca Malentacchi, Maria Grazia Colao, Gian Maria Rossolini, Costanza Fiorelli, Daniela Massi, Alberto Antonelli, Sara Cuffari, Trentina Di Muccio, Alessandro Bartoloni, Michele Spinicci, Lorenzo Zammarchi

**Affiliations:** 1 Department of Experimental and Clinical Medicine, University of Florence, Largo Brambilla 3, 50134 Florence, Italy; anna.barbiero@unifi.it (A.B.); andrea.aiello@unifi.it (A.A.); simona.pollini@unifi.it (S.P.); gianmaria.rossolini@unifi.it (G.M.R.); alberto.antonelli@unifi.it (A.A.); sara.cuffari@unifi.it (S.C.); alessandro.bartoloni@unifi.it (A.B.); michele.spinicci@unifi.it (M.S.); 2 Microbiology and Virology Unit, Careggi University Hospital, Largo Brambilla 3, 50134, Florence, Italy; cicconen@aou-careggi.toscana.it (N.C.); malentacchif@aou-careggi.toscana.it (F.M.); colaog@aou-careggi.toscana.it (M.G.C.); 3 Infectious and Tropical Diseases Unit, Careggi University Hospital, Largo Brambilla 3, 50134 Florence, Italy; fiorellic@aou-careggi.toscana.it; 4 Section of Pathology, Department of Health Sciences, University of Florence, 50121 Florence, Italy; daniela.massi@unifi.it; 5 Department of Infectious Diseases, Istituto Superiore di Sanità, Viale Regina Elena 299, 00161 Rome, Italy; trentina.dimuccio@iss.it

**Keywords:** leishmaniasis, cutaneous, Europe, Italy, swabs, neglected tropical diseases, vector-borne diseases

## Abstract

Background: The case confirmation of CL relies on the direct demonstration of the parasite in clinical specimens from skin tissues. Despite most research efforts focusing on biopsy samples as the preferred diagnostic specimen for the detection of *Leishmania* spp., the use of non-invasive sampling, such as cutaneous swabs, combined with the use of molecular assays, has shown promising results. Methods: We conducted a retrospective study aimed at comparing the performance of different invasive and non-invasive diagnostic techniques, employed for the diagnosis of CL, in an Italian tertiary care center. Results: We observed 29 cases of CL between 2008 and June 2024. Considering the demonstration of *Leishmania* spp. on culture, biopsy PCR, histology, or smear microscopy as the reference diagnostic test for CL, molecular assays on cutaneous swabs showed a sensitivity of 100% (95% C.I. 73.5–100). Overall, PCR performed on swab specimens allowed for the detection of three cases that biopsy histology (in two cases) and microscopic examination of cutaneous smear (in three cases) would have failed to identify. Conclusion: Non-invasive swab sampling, combined with molecular analysis, can be a valuable tool for a more accessible and patient-friendly diagnostic approach for CL. Should our preliminary results be confirmed, this test could become the first-line diagnostic tool for CL, reserving biopsy as a second-level test or for cases in which the differential diagnosis includes malignancy or other concerning diseases. Further studies aimed at defining the efficiency of this diagnostic method and providing standardized diagnostic protocols would be needed to provide stronger evidence supporting its recommendation.

## 1. Introduction

Leishmaniasis consists of a group of diseases transmitted by phlebotomine sandflies of the *Psychodidae* family and caused by protozoans of the genus *Leishmania* spp.

It can present with a wide clinical spectrum that includes visceral and tegumentary forms. The only *Leishmania* species known to be endemic in Southern Europe is *L. infantum*, which may be responsible for visceral leishmaniasis (VL), cutaneous leishmaniasis (CL). and, less frequently, mucocutaneous leishmaniasis (ML) [1,2,3]. While VL is associated with high morbidity and mortality if left untreated, tegumentary forms (CL and ML) usually present with more indolent evolution. However, mucosal involvement can compromise respiratory and phonatory function, with possible evolution towards life-threatening and severely destructive outcomes. Moreover, CL can evolve into disfiguring and dysfunctional scars. For these reasons, tegumentary leishmaniasis has a significant impact on affected subjects in terms of stigmatization and quality of life, especially in endemic low- and middle-income countries [4].

Tegumentary leishmaniasis is the most common clinical form of human leishmaniasis worldwide [5]; it is estimated that 0.6–1 million people are affected globally by tegumentary leishmaniasis and the global burden is likely increasing [6]. The number of imported cases of CL has increased in many countries of Western Europe, associated with climate and environmental changes, but also increasing travel, ecotourism activity, military operations, and immigration [7]. In Italy, southern regions, the big islands (Sardinia and Sicily), the Tyrrhenian coast, and, to a lesser extent, the Adriatic coast are well-known endemic regions for human leishmaniasis [8,9,10,11,12,13,14,15]. More recently, an increasing number of cases has been recorded also in North-Eastern regions and Central Italy [16,17,18,19,20]. This shifting epidemiological pattern suggests that the disease could be of increasing interest in the upcoming future of our country.

The diagnosis of CL is challenging, due to very diverse clinical presentation and a broad spectrum of differential diagnosis. However, prompt diagnosis is important to limit disease progression, mucosal involvement, and the development of disfiguring or dysfunctional scars [21]. The case confirmation of CL relies on the direct demonstration of the parasite in clinical specimens of skin tissues, using microscopic examination or molecular analysis [22]. Most of the available literature on the detection of *Leishmania* DNA has focused on biopsy samples for a long time, while a few studies have evaluated the use of non-invasive sampling methods, such as tape discs, cytology brushes, or swabs [22,23,24,25]. Less invasive diagnostic approaches, combined with the implementation of molecular assays, have gained increasing interest in recent years worldwide and proved to be highly accurate, specific, and sensitive [23,24,25,26]. However, such reports are highly heterogeneous, and standardized PCR-based diagnostic protocols on non-invasive samples for CL diagnosis, although attractive, are currently lacking [24]. Furthermore, although several studies on the utility of swab PCR as a diagnostic tool for CL have been conducted in endemic areas of Latin America, Sub-Saharan Africa, and South-East Asia [24,27,28], poor information has been provided from the Mediterranean area. To our knowledge, the use of PCR-based swab sampling for CL diagnosis in Europe has only sporadically been reported, and biopsy still represents the standard diagnostic procedure for CL; as regards Italy, studies on the diagnostic performance of the molecular detection of *Leishmania* spp. on superficial skin specimens have not yet been conducted [25,26,28,29,30]. Given the geography-dependent characteristics of CL (i.e., different causative species, species-dependent parasite load, and lesion presentation and evolution), further research on the efficiency of non-invasive diagnostic approaches is needed in Southern Europe [31].

We conducted a retrospective study aimed at analyzing and comparing the performance of different invasive and non-invasive diagnostic techniques, employed for the diagnosis of CL, in an Italian tertiary care center over a 16-year period.

## 2. Materials and Methods

We conducted a retrospective study at the Infectious and Tropical Diseases Unit, Careggi University Hospital (Florence, Italy), between 2008 and June 2024. We included all patients diagnosed with CL based on clinical features and direct detection of *Leishmania* spp. in skin lesions through at least one of the following tests: skin biopsy with histological exam, microscopic examination of cutaneous smear stain, PCR on skin biopsy, PCR on lesion swab, and culture on biopsy specimens. Of note, cultural exams were performed at the Istituto Superiore di Sanità in Rome (ISS). Positive serology alone was not considered sufficient for CL diagnosis if not accompanied by a direct microbiological demonstration of *Leishmania* spp. on a cutaneous lesion. All enrolled patients were treated and followed up in the enrolling center.

By consulting digital records of both inpatients and outpatients, we collected the clinical and demographic data of enrolled subjects. Data about microbiological assays and pathology results were gathered.

Clinical, epidemiological, and demographic information of the observed cases was collected using REDCap 8.11.6. (Project REDCap, USA).

We defined autochthonous episodes as those where the likely site of infection was within Italy, whereas imported cases were those where the infection was most likely acquired outside Italy.

The definition of simple and complex forms of CL was based on the criteria of the IDSA/ASTMH guidelines [23].

### 2.1. Swab Sampling

In the case of ulcerated lesions and the presence of an eschar, after disinfection with sodium hypochlorite, it was softened by applying a wrap with sterile saline solution on the lesion for 5 to 10 min. Thereafter, the eschar (or part of it) was removed, thus uncovering the underlying infected dermal tissues.

In the case of non-ulcerated papules or dry skin lesions, we scraped the most superficial skin layers in order to uncover the underlying superficial skin tissues.

Afterwards, the swab (Copan eSwab, Copan Italia, Brescia, Italy) was rubbed in a clockwise direction over the lesion surface (especially on the margins), allowing the collection of material from the exudating skin lesion. The specimen was then preserved at room temperature and reached the microbiology laboratory within the next 6 h after collection. Molecular analysis on fresh swab specimens was therefore performed right after collection.

### 2.2. Molecular Assays

Polymerase Chain Reaction (PCR) was performed using a qualitative Real-Time PCR kit (Clonit SRL, Milan, Italy) targeting the 18S ribosomal RNA sequences of *Leishmania* genome. DNA extraction and Real-Time PCR were performed on the automated ELITe InGenius^®^ platform (Elitechgroup SAS, Puteaux, France). Briefly, DNA was extracted from 200 µL of swab transport medium or the biopsic material, using the ELITe InGenius^®^ SP 200 extraction reagents according to manufacturer specifications, and PCR was performed using the *Leishmania* spp. qualitative Real-Time PCR kit on the built-in ELITe InGenius^®^ thermal cycler. The PCR results were analyzed by the ELITe InGenius^®^ dedicated software.

*Leishmania* spp. identification was performed either at the Microbiology and Virology laboratory at Careggi University Hospital, or at the ISS, by sequencing a segment of the rRNA internal transcribed spacer 2 (ITS2, using LGITSF2 5′-GCATGCCATATTCTCAGTGTC-3′ and LGITSR2 5′-GGCCAACGCGAAGTTGAATTC-3′ oligonucleotides) or a fragment of Heat Shock Protein-70 gene for New World *Leishmania* spp. (Hsp-70 F-fragment, using F25 5′-GGACGCCGGCACGATTKCT-3′ and R1310 5′-CCTGGTTGTTGTTCAGCCACTC-3′ oligonucleotides), as previously described [32,33].

### 2.3. Serology

Serological tests were performed using two different commercial kits. The Leishmania-Spot IF (bioMérieux, Marcy-l’Étoile, France), based on indirect immunofluorescence (IF), was used until 2021. Thereafter, serologies were performed using the Leishmania VirClia^®^ IgG-IgM Monotest (Vircell, Granada, Spain), based on chemiluminescent immunoassay (CLIA), following the manufacturers’ instructions.

### 2.4. Culture

Fresh material was seeded in Evan’s Modified Tobie’s Medium (EMTM) and ‘Sloppy Evans’ [34], and cultures were checked for promastigote growth up to 30 days. *Leishmania*-positive cultures were cryopreserved at ISS. Genomic DNA extraction from promastigote cultures was performed using the DNA extraction protocol of the Maxwell^®^ 16 Cell DNA Purification Kit (Promega, Madison, WI, USA) following the manufacturer’s instructions. The DNA was stored at −20 °C until use.

### 2.5. Statistical Analysis

A descriptive analysis was conducted to analyze clinical and demographic characteristics of the enrolled population. Median and interquartile rates (IQRs) were applied to describe continuous variables, while categorical variables were described through frequencies and proportions.

The association between categorical variables was evaluated with the Chi-square test or Fisher’s exact test where appropriate, and continuous variables with the Wilcoxon rank-sum test. Results were considered statistically significant for *p*-values < 0.05. The software STATA v18.0 (STATACorp, USA) was used for descriptive statistical analyses.

### 2.6. Ethical Considerations

The enrolled patients gave informed consent for participation to this study, and each record was pseudonymized before the compilation of the Case Report Form (CRF). The study was performed in accordance with the ethical principles of the Declaration of Helsinki and with the International Conference on Harmonization Good Clinical Practice guidelines. Data collection was approved by the local Ethics Committee “Comitato Etico Regione Toscana—Area Vasta Centro” (protocol code: TOSMANIA_2023; approved on 27 February 2024; amendment approved on 18 March 2025; registration code: 25425_oss/Em. 2025-021 ID 25425_oss).

## 3. Results

Between 2008 and June 2024, we observed 29 cases of CL diagnosed by at least one direct microbiological assay. Twenty-two out of the twenty-nine (75.9%) patients were males. Median age was 42 (IQR: 34–62). As regards the origin of infection, we observed 19 (65.5%) autochthonous and 9 (31.0%) imported cases, while information on infection origin was not available for 1 case. Of the imported cases, one out of nine was imported from Palestine and seven out of nine were imported from Central and South America (Mexico (two), Ecuador (one), Argentina (one), Nicaragua (one), and Peru (two)). The place of infection could not be determined in one imported case due to multiple countries of exposure.

The median number of skin lesions was 2 (IQR: 1–2) with a median size of 2.3 cm (IQR: 1.5–3.3).

Information regarding the clinical presentation was available for 26/29 patients, with 13/26 (50.0%) patients presenting with simple forms of CL, and 13/26 (50.0%) fulfilling the criteria for complex CL. There was no statistically significant difference in the distribution of simple and complex CL based on the origin of the infection (46.2% complex forms among autochthonous vs. 53.9% among imported cases; *p* = 0.141).

The most frequent criterion for complex CL was the presence of lesions involving the face (including ears, eyelids, or lips), fingers, toes, other joints, or genitalia, in 7/13 (53.9%) cases. Less common criteria were the presence of a skin lesion with diameter > 5 cm (4/13, 30.8%), infection acquired in the “mucosal belt” area [23] (4/13, 30.8%), having more than four lesions with a diameter > 1 cm (3/13, 23.1%), an immunocompromised host (2/13, 15.4%), the unfeasibility of local treatment (1/13, 7.7%), and the presence of regional lymphadenopathy (1/13, 7.7%).

The median time to diagnosis was 128 days (IQR: 102–181), with a significantly longer diagnostic delay for autochthonous forms, compared to the imported cases (177 vs. 107 days, respectively; *p* = 0.0302).

Not all diagnostic tests were performed in all subjects. *Leishmania* PCR on cutaneous swab was the most frequently performed test, with 20/29 (69.0%) tested subjects, followed by skin biopsy histological exam (17/29, 58.6%), microscopy on cutaneous smear stain (10/29, 34.5%), PCR on skin biopsy (4/29, 13.8%), and *Leishmania* cultural exam on biopsy specimens (3/29, 10.3%). Overall, 18/29 (62.1%) patients underwent biopsy. *Leishmania* spp. identification through genotyping was performed for seven imported and three autochthonous cases: *L. mexicana* (one), *L. braziliensis* (one), *L. panamensis* (three), and *L. major* (two) were identified in imported cases of CL, whereas *L. infantum* (two) and *L. donovani* complex (one) were identified in autochthonous cases. Genotyping was performed on swab samples in five cases and on biopsy samples in five cases.

When comparing the results obtained for each diagnostic assay in the case of a clinically confirmed diagnosis of CL (i.e., suggestive clinical presentation and response to anti-leishmanial therapies, in the absence of other possible diagnoses), molecular assays on cutaneous swabs or biopsy specimens and *Leishmania* culture on biopsies had the highest proportion of positive results (100%) (Table 1).

The two cases with negative histology and the three with negative cutaneous smear stain were all positive to cutaneous swab PCR (Table 2). Eleven out of twenty-nine patients did not undergo biopsy and were diagnosed through positive PCR on lesions swab, supported by compatible clinical features (e.g., clinical presentation, exclusion of other causes, and response to specific therapies) and/or positive microscopical examination on scarping smears. Details of the diagnostic tests used for each enrolled patient and their results are reported in Table 2.

Among the 21 patients who had a direct demonstration of *Leishmania* spp. through culture, biopsy PCR, histology, or smear microscopy, 12/21 also performed Leishmania PCR on cutaneous swabs, with 12/12 positive results. If the demonstration of *Leishmania* spp. on culture, biopsy PCR, histology, or smear microscopy is considered as the reference test, the swab PCR showed a 100% sensitivity (95% C.I. 73.5–100).

Specificity could not be calculated for any of the diagnostic assays due to the study design which did not include a negative control group.

## 4. Discussion

This study shows the clinical characteristics of a CL cohort observed in a tertiary care center over 16 years, with a focus on the performance of different diagnostic tests.

Between 2008 and June 2024, we observed 29 cases of CL. Coherently with data reported in the literature [26,35], males were predominant, representing the 75.9% of the study population.

We observed a median age of 42 (IQR: 34–62) years, consistently with other data reported in Italy for CL [36]. While VL can frequently affect the older and immunosuppressed population, CL frequently affects younger people [37,38]. This likely reflects a higher risk of exposure in this age group due to outdoor activities for professional or recreational reasons.

According to clinical history and epidemiological investigation, most of the observed infections were acquired in Italy (65.5%), whereas most imported cases (seven out of nine) came from Central and South America; special attention should be paid in the case of cutaneous lesion appearance after traveling to these endemic areas. Prompt diagnosis, species identification, and appropriate management are particularly important in the case of tegumentary leishmaniasis acquired in South America and especially in the “mucosal belt” area. Indeed, infection caused by species related with mucosal involvement is common in this region, and, given its possibly severe clinical implications, it needs to be rapidly ruled out or managed if already present [23]. For this reason, *Leishmania* spp. genotyping was performed for all the imported cases but two, for which collected samples were not sufficient to perform further molecular study and good clinical response to systemic specific therapy was documented. Genotyping was also performed for three autochthonous cases due to an unclear epidemiological history, in order to confirm infection origin and the infecting species. Although stronger evidence is needed, the results from this study suggest that the application of molecular methods not only on biopsy specimens but also on superficial swabs is efficient for the identification of different Old- and New-World *Leishmania* species.

In total, 50.0% of enrolled patients fulfilled the criteria for complex CL, with no difference in the distribution of complex forms between imported and autochthonous infections. As suggested by these results, despite Old-World CL usually being associated with a less severe clinical course, it can frequently meet the criteria for complex CL as defined by IDSA/ASTMH, therefore determining a non-negligible clinical impact and often requiring systemic treatment [23].

A median time of 128 (IQR: 102–181) days was needed for the diagnosis of CL, being significantly longer in the case of autochthonous infections, compared to the imported cases. This underlines the limited awareness among patients, who are not informed about the presence and the clinical presentation of the disease, but also among clinicians, who often do not take CL into consideration among the differential diagnoses of chronic cutaneous lesions, especially at our latitudes.

Both biopsic and superficial sampling through scraping and the use of cutaneous swabs were employed in this study for the diagnosis of CL. Cutaneous swab PCR was the most frequently performed diagnostic technique, and, when both swab PCR and other direct tests were performed, it showed a 100% sensitivity (95% C.I. 73.5–100), if the direct demonstration of *Leishmania* spp. through culture, biopsy PCR, histology, or smear microscopy is considered as the reference diagnostic test. Swab PCR allowed for a non-invasive diagnosis for 11/29 patients whose diagnostic workup did not require biopsy. Moreover, it allowed the detection of three cases of CL that biopsy (in two cases) and skin smear (in three cases) would have failed to identify, highlighting that this test may increase the diagnostic yield in CL.

**Figure 1:**
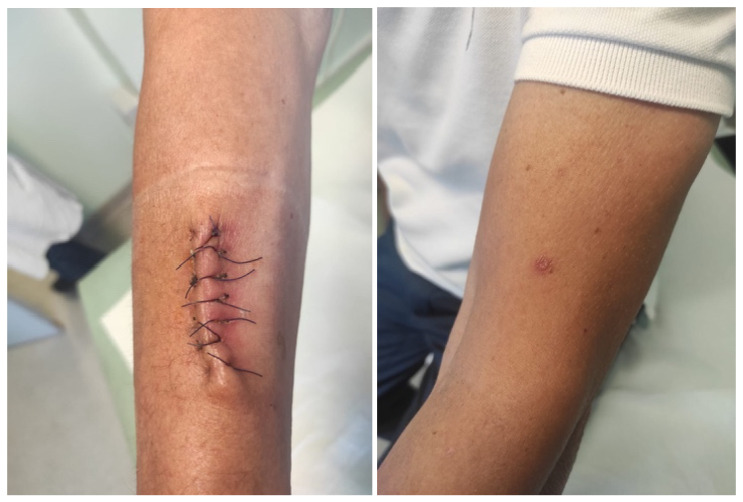
Residual eschar after performing skin biopsy (**left**) and swab PCR (**right**) for the diagnosis of CL on two different lesions of the same patient (biopsy was performed prior to evaluation in our outpatient clinic, where only the skin swab PCR was performed).

In line with data in the literature, our results confirm that PCR-based techniques for CL diagnosis have great sensitivity compared to non-molecular diagnostic methods [27,39]. Moreover, this study sheds light on the possible role of the application of molecular methods to superficial swab sampling as a possible first-line diagnostic step for CL. Although the diagnosis of CL is still often based on invasive sampling in the clinical European context, molecular techniques allow an increasing use of more patient-friendly approaches, and swab collection is emerging as a powerful diagnostic tool because of its non-invasive and simple collection method [28,40]. Previous studies reported promising results, with the sensitivity of PCR-based diagnosis through skin swab collection reaching 93–98% [27,39]. A study conducted in Colombia by Adams et al., comparing different molecular extraction methods combined with swab or skin aspirate sampling for CL diagnosis, showed that swab sampling combined with standardized DNA extraction was the most efficient method for *Leishmania* DNA detection, with higher sensitivity (98%; 95% CI: 91–100%) and specificity (84%; 95% CI: 64–95%) compared to aspirated material [27]. Coherently, Gomes et al. observed that swabs and biopsy specimens have similar sensitivity and accuracy for the diagnosis of American tegumentary leishmaniasis [30]. As regards Europe, a retrospective study conducted in a referral center in Barcelona in 2021 reported that molecular assays for *Leishmania* DNA detection were 100% concordant when biopsy and skin swab were performed simultaneously [26]. However, molecular methods combined with swab sampling are not widely used in clinical practice in the European setting, due to a lack of stronger evidence and standardized protocols, and this diagnostic approach is not supported by national and international guidelines. Compared to biopsy specimen collection, swab PCR shows several advantages such as being painless and easy to perform. In addition, it does not cause residual scars (Figure 1), and it offers the possibility of being performed in any outpatient setting, including those with limited resources [25,27]. Conversely, despite full-thickness skin biopsy being often considered the most robust sample for the diagnosis of CL [31,41], for it to be correctly performed, its collection requires that the clinician has certain experience, and the sampling itself can be time-expensive in an outpatient clinic. Moreover, it should be considered that *Leishmania* parasites are unevenly distributed on a skin lesion, thus determining variable sampling sensitivity, depending on the specific lesion site where the tissue specimen is collected [28]. On the contrary, swab sampling would allow multiple samplings in different lesion sites, therefore improving the diagnostic performance.

Despite the routine employment of molecular assays on swab specimens in our center, biopsy collection was still performed in 62.1% of the study population. In most cases, biopsy was performed before accessing our outpatient clinic, and patients were referred to us for CL management following biopsy result. On one hand, these findings underline that this diagnostic method is still widely accepted by clinicians as a first-step approach; on the other hand, it could represent the final tentative to investigate a lesion of unknown origin, after a number of inconclusive diagnostic and therapeutic approaches, as supported by the considerable diagnostic delay in our cohort.

Within the limitations of this study, the absence of an approved procedure for molecular diagnosis on non-invasive samples should be noted; indeed, the procedure, which is routinely used for leishmaniasis diagnosis on blood samples, still lacks standardization on cutaneous samples. However, this molecular approach also proved highly efficient in identifying the presence of *Leishmania* genome in skin swabs. Considering this, the standardization of PCR-based assays applied to non-invasive samples and consensus diagnostic protocols between different centers would improve diagnostic workflow and could result in comparable results, particularly in the Mediterranean area [40]. Another limitation is that the specificity of the employed diagnostic methods could not be determined due to the study design. Moreover, although no confirmed CL cases in this study had a negative swab PCR result, stronger evidence is needed to define this technique’s sensitivity. Given that the success of molecular methods depends heavily on the quality of DNA extraction and the parasite load, the swab might not capture enough parasites for detection, leading to false negatives. The risk of false-positive results has also been addressed in previous studies and could be raised by extremely sensitive molecular reactions [42]. The possible role of contamination in determining false-positive results should also be assessed. In this study, all reported cases of CL had a confirmed diagnosis either through other diagnostic methods or ex juvantibus (i.e., through the observation of lesion regression after anti-leishmanial therapy), thus mitigating the possibility of false-positive results.

## 5. Conclusions

The research of newer and more acceptable methods for the diagnosis of CL is of increasing interest worldwide. The results of this study suggest that non-invasive swab sampling, combined with molecular analysis, can be a valuable tool for a more patient-friendly, time-effective, and easy-to-perform diagnostic approach for CL. We hypothesize that this test could become the first-line diagnostic test for CL, reserving biopsy as a second-level test or for cases in which the differential diagnosis is difficult and includes malignancy or other concerning diseases.

Further studies aimed at defining the sensitivity, specificity, and accuracy of less invasive diagnostic techniques for CL would be needed, in order to provide stronger evidence supporting its recommendation. Finally, more research effort would be needed to provide standardized and homogenized diagnostic protocols for CL diagnosis through non-invasive sampling.

## Figures and Tables

**Table 1:** Number of tested subjects, positive results, and test sensitivity for each diagnostic test performed for the diagnosis of autochthonous and imported cases of CL.

	No. Performed Tests	No. Positive Tests	% of Positive Results/Performed Tests (95% C.I.)
Cutaneous swab PCR	20	20	100% (83.2–100)
Skin biopsy histology	17	15	88.2% (63.6–98.5)
Cutaneous smear stain	10	7	70.0% (34.8–93.3)
Skin biopsy PCR	4	4	100% (39.8–100)
Leishmania culture on biopsy	3	3	100% (29.2–100)

**Table 2:** Tests performed and results obtained for each enrolled patient diagnosed with CL according to clinical features and direct detection of *Leishmania* spp. in skin lesions.

Patient No.	Swab PCR	Scraping Smear	PCR on Biopsy	Histology	Culture *	Serology
1	positive	NA	NA	NA	NA	negative
2	positive	positive	NA	NA	NA	NA
3	positive	positive	NA	NA	NA	negative
4	positive	positive	NA	NA	NA	positive
5	positive	negative	NA	negative	NA	NA
6	positive	NA	NA	NA	NA	NA
7	positive	NA	NA	NA	NA	negative
8	positive	NA	NA	NA	NA	NA
9	positive	negative	NA	negative	NA	negative
10	positive	positive	NA	NA	NA	negative
11	positive	NA	NA	NA	NA	positive
12	positive	NA	NA	NA	NA	NA
13	positive	NA	NA	positive	NA	NA
14	positive	positive	NA	positive	NA	negative
15	positive	NA	NA	positive	NA	NA
16	NA	NA	positive	positive	NA	negative
17	NA	NA	positive	positive	NA	NA
18	NA	NA	NA	positive	NA	NA
19	NA	NA	NA	positive	NA	negative
20	positive	NA	NA	NA	positive	positive
21	positive	negative	NA	positive	NA	NA
22	NA	NA	NA	positive	NA	positive
23	positive	NA	positive	positive	positive	negative
24	NA	positive	positive	positive	positive	negative
25	positive	NA	NA	positive	NA	positive
26	NA	NA	NA	positive	NA	NA
27	NA	NA	NA	positive	NA	positive
28	NA	NA	NA	positive	NA	NA
29	positive	positive	NA	NA	NA	NA

NA = not available. * Culture was performed on biopsic specimens.

## Data Availability

The data presented in this study are available on request from the corresponding author due to privacy reasons.

## References

[1] Guery R., Walker S.L., Harms G., Neumayr A., Van Thiel P., Gangneux J.-P., Clerinx J., Söbirk S.K., Visser L., Lachaud L. (2021). Clinical diversity and treatment results in Tegumentary Leishmaniasis: A European clinical report in 459 patients. PLoS Negl. Trop. Dis..

[2] Bruno F., Vitale F., La Russa F., Reale S., Späth G.F., Oliveri E., Gargano V., Valenza V., Facciponte F., Giardina S. (2022). Retrospective Analysis of Leishmaniasis in Sicily (Italy) from 2013 to 2021: One-Health Impact and Future Control Strategies. Microorganisms.

[3] Maia C., Conceição C., Pereira A., Rocha R., Ortuño M., Muñoz C., Jumakanova Z., Pérez-Cutillas P., Özbel Y., Töz S. (2023). The estimated distribution of autochthonous leishmaniasis by Leishmania infantum in Europe in 2005–2020. PLoS Negl. Trop. Dis..

[4] Kassi M., Kassi M., Afghan A.K., Rehman R., Kasi P.M. (2008). Marring leishmaniasis: The stigmatization and the impact of cutaneous leishmaniasis in Pakistan and Afghanistan. PLoS Negl. Trop. Dis..

[5] Alvar J., Vélez I.D., Bern C., Herrero M., Desjeux P., Cano J., Jannin J., den Boer M., WHO Leishmaniasis Control Team (2012). Leishmaniasis worldwide and global estimates of its incidence. PLoS ONE.

[6] Leishmaniasis. https://www.who.int/news-room/fact-sheets/detail/leishmaniasis.

[7] Van der Auwera G., Davidsson L., Buffet P., Ruf M.-T., Gramiccia M., Varani S., Chicharro C., Bart A., Harms G., Chiodini P.L. (2022). Surveillance of leishmaniasis cases from 15 European centres, 2014 to 2019: A retrospective analysis. Eurosurveillance.

[8] Bettini S., Pampiglione S., Maroli M. (1977). Studies on Mediterranean leishmaniasis: V.A. preliminary epidemiological survey of human leishmaniasis in Tuscany. Trans. R. Soc. Trop. Med. Hyg..

[9] Bettini S., Pozio E., Gradoni L. (1980). Leishmaniasis in Tuscany (Italy): (II) Leishmania form wild Bodentia and Carnivora in a human and canine leishmaniasis focus. Trans. R. Soc. Trop. Med. Hyg..

[10] Gradoni L., Pozio E., Bettini S., Gramiccia M. (1980). Leishmaniasis in Tuscany (Italy). (III) The prevalence of canine leishmaniasis in two foci of Grosseto Province. Trans. R. Soc. Trop. Med. Hyg..

[11] Bettini S., Maroli M., Gradoni L. (1981). Leishmaniasis in Tuscany (Italy): (IV) An analysis of all recorded human cases. Trans. R. Soc. Trop. Med. Hyg..

[12] Pozio E., Gradoni L., Bettini S., Gramiccia M. (1981). Leishmaniasis in Tuscany (Italy): VI. Canine leishmaniasis in the focus of Monte Argentario (Grosseto). Acta Trop..

[13] Gradoni L., Pozio E., Gramiccia M., Maroli M., Bettini S. (1983). Leishmaniasis in Tuscany (Italy): VII. Studies on the role of the black rat, Rattus rattus, in the epidemiology of visceral leishmaniasis. Trans. R. Soc. Trop. Med. Hyg..

[14] Bettini S., Gramiccia M., Gradoni L., Pozio E., Mugnai S., Maroli M. (1983). Leishmaniasis in Tuscany (Italy): VIII. Human population response to leishmanin in the focus of Monte Argentario (Grosseto) and epidemiological evaluation. Ann. Parasitol. Hum. Comp..

[15] Gramiccia M., Scalone A., Di Muccio T., Orsini S., Fiorentino E., Gradoni L. (2013). The burden of visceral leishmaniasis in Italy from 1982 to 2012: A retrospective analysis of the multi-annual epidemic that occurred from 1989 to 2009. Euro Surveill..

[16] Franceschini E., Puzzolante C., Menozzi M., Rossi L., Bedini A., Orlando G., Gennari W., Meacci M., Rugna G., Carra E. (2016). Clinical and Microbiological Characteristics of Visceral Leishmaniasis Outbreak in a Northern Italian Nonendemic Area: A Retrospective Observational Study. BioMed Res. Int..

[17] Defilippo F., Carrera M., Lelli D., Canziani S., Moreno A., Sozzi E., Manarolla G., Chiari M., Marco F., Cerioli M.P. (2022). Distribution of Phlebotomine Sand Flies (Diptera: Psychodidae) in the Lombardy Region, Northern Italy. Insects.

[18] Varani S., Cagarelli R., Melchionda F., Attard L., Salvadori C., Finarelli A., Gentilomi G., Tigani R., Rangoni R., Todeschini R. (2013). Ongoing outbreak of visceral leishmaniasis in Bologna Province, Italy, November 2012 to May 2013. Eurosurveillance.

[19] Innocenti F., Gemmi F., Voller F., Barnini S., Gemignani G., Cosma C., Del Riccio M., Alderotti G., Arzilli G., Rizzo C. (2023). La Sorveglianza Epidemiologica Delle Malattie Infettive in Toscana 2022.

[20] Barbiero A., Spinicci M., Aiello A., Maruotto M., Antonello R.M., Formica G., Piccica M., Isola P., Parisio E.M., Nardone M. (2024). The Uprise of Human Leishmaniasis in Tuscany, Central Italy: Clinical and Epidemiological Data from a Multicenter Study. Microorganisms.

[21] Control of the Leishmaniases WHO TRS N°949. https://www.who.int/publications-detail-redirect/WHO-TRS-949.

[22] Manual on Case Management and Surveillance of the Leishmaniases in the WHO European Region. https://www.who.int/publications/i/item/9789289052511.

[23] Aronson N., Herwaldt B.L., Libman M., Pearson R., Lopez-Velez R., Weina P., Carvalho E.M., Ephros M., Jeronimo S., Magill A. (2016). Diagnosis and Treatment of Leishmaniasis: Clinical Practice Guidelines by the Infectious Diseases Society of America (IDSA) and the American Society of Tropical Medicine and Hygiene (ASTMH). Clin. Infect. Dis. Off. Publ. Infect. Dis. Soc. Am..

[24] Galluzzi L., Ceccarelli M., Diotallevi A., Menotta M., Magnani M. (2018). Real-time PCR applications for diagnosis of leishmaniasis. Parasit. Vectors.

[25] Boni S.M., Oyafuso L.K., Soler R.d.C., Lindoso J.A.L. (2017). Efficiency of noninvasive sampling methods (swab) together with Polymerase Chain Reaction (PCR) for diagnosing American Tegumentary Leishmaniasis. Rev. Inst. Med. Trop. Sao Paulo.

[26] Silgado A., Armas M., Sánchez-Montalvá A., Goterris L., Ubals M., Temprana-Salvador J., Aparicio G., Chicharro C., Serre-Delcor N., Ferrer B. (2021). Changes in the microbiological diagnosis and epidemiology of cutaneous leishmaniasis in real-time PCR era: A six-year experience in a referral center in Barcelona. PLoS Negl. Trop. Dis..

[27] Adams E.R., Gomez M.A., Scheske L., Rios R., Marquez R., Cossio A., Albertini A., Schallig H., Saravia N.G. (2014). Sensitive diagnosis of cutaneous leishmaniasis by lesion swab sampling coupled to qPCR. Parasitology.

[28] van Henten S., Kassa M., Fikre H., Melkamu R., Mekonnen T., Dessie D., Mulaw T., Bogale T., Engidaw A., Yeshanew A. (2024). Evaluation of Less Invasive Sampling Tools for the Diagnosis of Cutaneous Leishmaniasis. Open Forum Infect. Dis..

[29] Deepachandi B., Weerasinghe S., Soysa P., Karunaweera N., Siriwardana Y. (2019). A highly sensitive modified nested PCR to enhance case detection in leishmaniasis. BMC Infect. Dis..

[30] Gomes C.M., Cesetti M.V., de Paula N.A., Vernal S., Gupta G., Sampaio R.N.R., Roselino A.M. (2017). Field Validation of SYBR Green- and TaqMan-Based Real-Time PCR Using Biopsy and Swab Samples To Diagnose American Tegumentary Leishmaniasis in an Area Where Leishmania (Viannia) braziliensis Is Endemic. J. Clin. Microbiol..

[31] Merino-Espinosa G., Rodríguez-Granger J., Morillas-Márquez F., Tercedor J., Corpas-López V., Chiheb S., Alcalde-Alonso M., Azaña-Defez J.M., Riyad M., Díaz-Sáez V. (2018). Comparison of PCR-based methods for the diagnosis of cutaneous leishmaniasis in two different epidemiological scenarios: Spain and Morocco. J. Eur. Acad. Dermatol. Venereol..

[32] de Almeida M.E., Steurer F.J., Koru O., Herwaldt B.L., Pieniazek N.J., da Silva A.J. (2011). Identification of Leishmania spp. by Molecular Amplification and DNA Sequencing Analysis of a Fragment of rRNA Internal Transcribed Spacer 2. J. Clin. Microbiol..

[33] van der Auwera G., Bart A., Chicharro C., Cortes S., Davidsson L., Di Muccio T., Dujardin J.-C., Felger I., Paglia M.G., Grimm F. (2016). Comparison of Leishmania typing results obtained from 16 European clinical laboratories in 2014. Euro Surveill..

[34] Taylor A.E.R., Baker J.R. (1987). In Vitro Methods for Parasite Cultivation.

[35] Garrido-Jareño M., Sahuquillo-Torralba A., Chouman-Arcas R., Castro-Hernández I., Molina-Moreno J.M., Llavador-Ros M., Gómez-Ruiz M.D., López-Hontangas J.L., Botella-Estrada R., Salavert-Lleti M. (2020). Cutaneous and mucocutaneous leishmaniasis: Experience of a Mediterranean hospital. Parasit. Vectors.

[36] Gaspari V., Gritti T., Ortalli M., Santi A., Galletti G., Rossi A., Rugna G., Mattivi A., Matteo G., Belloli G.L. (2022). Tegumentary Leishmaniasis in Northeastern Italy from 2017 to 2020: A Neglected Public Health Issue. Int. J. Environ. Res. Public Health.

[37] Herrador Z., Gherasim A., Jimenez B.C., del sol Granados M., Martín J.V.S., Aparicio P. (2016). Correction: Epidemiological Changes in Leishmaniasis in Spain According to Hospitalization-Based Records, 1997–2011: Raising Awareness towards Leishmaniasis in Non-HIV Patients. PLoS Negl. Trop. Dis..

[38] PAHO, WHO Visceral Leishmaniasis. https://www.paho.org/en/topics/leishmaniasis/visceral-leishmaniasis.

[39] Mimori T., Matsumoto T., Calvopiña M.H., Gomez E.A., Saya H., Katakura K., Nonaka S., Shamsuzzaman S.M., Hashiguchi Y. (2002). Usefulness of sampling with cotton swab for PCR-diagnosis of cutaneous leishmaniasis in the New World. Acta Trop..

[40] Torpiano P., Pace D. (2015). Leishmaniasis: Diagnostic issues in Europe. Expert Rev. Anti Infect. Ther..

[41] Vega-López F. (2003). Diagnosis of cutaneous leishmaniasis. Curr. Opin. Infect. Dis..

[42] Ovalle Bracho C., Porras de Quintana L., Muvdi Arenas S., Rios Parra M. (2007). Polymerase chain reaction with two molecular targets in mucosal leishmaniasis’ diagnosis: A validation study. Mem. Inst. Oswaldo Cruz.

